# Changes of Endothelin-1 and Calcitonin Gene-Related Peptide Concentrations in Patients with Cervical Radiculopathy after Wrist-Ankle Acupuncture-Moxibustion and Hot Compression with Chinese Herbal Medicine

**DOI:** 10.1155/2021/5433742

**Published:** 2021-12-18

**Authors:** Wei Li, Chi Yao, Yanghong Zhou, Sishi Chen

**Affiliations:** ^1^Department of Integrated Chinese and Western Medicine, Hunan Provincial People's Hospital, The First Affiliated Hospital of Hunan Normal University, Changsha, Hunan 410016, China; ^2^Department of Nephrology, Guangdong Province Traditional Chinese Medicine Hospital, Guangzhou, China

## Abstract

**Objective:**

This study aimed at investigating the effects of wrist-ankle acupuncture-moxibustion and hot compression with Chinese herbal medicine on pain symptoms, endothelin-1 (ET-1), and calcitonin gene-related peptide (CGRP) concentrations of patients with cervical radiculopathy (CR).

**Methods:**

A total of 82 patients with CR were randomly divided into the study group and control group, with 41 cases in each group. The control group was treated with standard treatment. In addition to standard treatment, the study group was additionally treated with wrist-ankle acupuncture-moxibustion and hot compression with Chinese herbal medicine. The ET-1 and CGRP concentrations in the plasma were measured by the radioimmunoassay method.

**Results:**

The total response rate in the study group and the control group was 97.55% and 82.93%, respectively. The study group showed lower scores of the visual analogue scale (VAS), Northwick Park Neck Pain Questionnaire (NPQ), McGill Pain Questionnaire (MPQ), numbness intensity assessment, and neck disability index (NDI) but higher scores of the Short Form-36 (SF-36) health survey questionnaire than the control group after treatment. Besides, the study group exhibited reduced ET-1 and substance P (SP) concentrations concomitant with increased CGRP and *β*-endorphin (*β*-EP) concentrations compared with the control group.

**Conclusion:**

Wrist-ankle acupuncture-moxibustion and hot compression with Chinese herbal medicine could effectively alleviate the pain of CR patients, affect ET-1 and CGRP concentrations, promote the recovery of cervical function, and improve the quality of life.

## 1. Introduction

Cervical radiculopathy (CR) is a common disorder which accounts for a high proportion of all cervical spondylosis, approximately 60 to 70% [1]. CR is characterized by a variety of clinical symptoms, such as neck and arm pain and numbness, nerve root dysfunction, and tendon reflex changes, mainly due to stimulation or compression in spinal nerve roots, which greatly affect people's lives and work [2]. In addition, CR is associated with increased healthcare costs and decreased productivity. It was estimated in 2010 that the healthcare costs and the value of lost productivity were $261 to $300 billion and $29.9 billion to $335 billion, respectively, caused by pain [3]. The occurrence of CR is prone to increase year by year due to aging of the population, lifestyle changes, and work or life pressure.

The diagnosis of CR should be confirmed by magnetic resonance imaging (MRI) or CT-combined clinical manifestations. In general, CR treatment involves surgical intervention, such as anterior cervical decompression and fusion, cervical disc replacement, and posterior foraminotomy, and nonsurgical intervention. All CR patients should be treated with strict conservative treatment for at least 6 weeks unless the occurrence of myelopathy or severe muscle weakness or no improvement after 6 to 12 weeks of nonsurgical intervention [4]. Numerous studies have indicated that nonsurgical treatment, consisting of anti-inflammatory drugs, physical therapy, cervical traction, epidural steroid injections, exercise, massage, acupuncture, etc., has been commonly used as an optimal scheme for most CR patients [2, 5]. It was observed that 75% to 90% of patients achieved symptom improvement through nonsurgical treatment [6].

Among nonsurgical treatments, acupuncture has been increasingly widely used as a comprehensive or supplementary therapy for pain, such as low back pain, knee osteoarthritis, headache, myofascial pain, neck pain, and fibromyalgia, due to its little risk of serious adverse reactions [7]. Additionally, moxibustion therapy [8] and hot compression with Chinese herbal medicine therapy [9] have been turned out to be an effective and safety way in alleviating pain pressure and improving the quality of life. Hot compression with Chinese herbal medicine is one of the traditional physical therapies of traditional Chinese medicine (TCM) and belongs to the category of external treatment. According to the treatment principle of TCM that treating cold syndrome with hot-natured drugs and the converse is also true, hot compression is mainly used to treat cold syndrome, wind-cold attacking, cold-dampness syndrome, or cold with blood stasis. Chinese herbal medicines used for hot compression consist of ginger, Ramulus Cinnamomi, and Herba Ephedrae for wind-cold attacking, dried ginger, cinnamon, *Poria cocos*, and *Alisma orientale* for cold-dampness syndrome, and Radix Aconiti, peach kernel, and safflower for cold with blood stasis. To confirm the effectiveness of wrist-ankle acupuncture-moxibustion and hot compression with Chinese herbal medicine in treating CR, the plasma concentrations of endothelin-1 (ET-1) and calcitonin gene-related peptide (CGRP) in patients with CR were examined as secondary outcome measures. Vertebral artery compression caused by cervical degeneration brings an increased transmural pressure followed by ischemia and hypoxia, and then endothelial cells release a large amount of ET-1 into blood, acting on the cerebellum and brainstem, leading to more severe ischemia and hypoxia in the vertebral artery [10]. CGRP, known as one of the strongest vasodilators so far, can strengthen myocardial contractility and expand blood vessels [11]. CGRP has a strong effect of relaxing isolated arterial rings, which is dependent of the endothelium. After removing the vascular endothelium, its relaxation effect disappears. In this study, 82 eligible patients with RC were selected as study subjects to explore the effects of wrist-ankle acupuncture-moxibustion and hot compression with Chinese herbal medicine on the pain symptoms, plasma ET-1 and CGRP concentrations, quality of life, and cervical vertebra function of patients with CR.

## 2. Materials and Methods

### 2.1. Eligible Study Subjects

From December 2019 to June 2020, 82 patients with CR treated in Guangdong Province Traditional Chinese Medicine Hospital were randomly assigned to the study group and control group with 41 cases each. The control group received standard treatment. In addition to standard treatment, the study group was supplemented with wrist-ankle acupuncture-moxibustion and hot compression with Chinese herbal medicine. All eligible participants should fulfill the following criteria: (a) positive Queckenstedt test examined by X-ray or magnetic resonance imaging (MRI), (b) occurrence of symptoms including neck muscle numbness and pain, widespread tenderness in the shoulder and neck, fatigue, trouble sleeping, and white-greasy tongue, and (c) good treatment compliance. Those patients are excluded if they meet any of the following criteria: (a) mental disorders, (b) important organ dysfunction, (c) severe skin disease or severe neck skin injury, (d) severe osteoporosis and congenital spinal stenosis or spinal cord injury, (e) cervical spinal tumors and osteomyelitis, (f) surgery history of cervical vertebrae, (g) cachexia patients, (h) women during the pregnant stage and breast-feed stage, and (i) intolerance to wrist-ankle acupuncture-moxibustion and hot compression with Chinese herbal medicine. This study was approved by the Ethics Committee of Guangdong Province Traditional Chinese Medicine Hospital, and the patients voluntarily participated and signed the informed consent form.

### 2.2. Treatment Protocols

Oral administration of Fenbid (National Medicine Permission no. H10900089, specification: 0.3 g × 20 tablets, SK&F Co., Ltd., China) and Sibelium (National Medicine Permission no. H10930003, specification: 5 mg × 20 tablets, Xian Janssen Pharmaceutical Ltd., China) was applied to the 41 patients in the control group. Twice a day for Fenbid and once a day for Sibelium were taken.

The remaining 41 cases in the study group additionally received wrist-ankle acupuncture-moxibustion and hot compression with Chinese herbal medicine. In brief, wrist-ankle acupuncture was carried out to the three points of wrists, involving the lateral edge of the radius, Waiguan (the midpoint of the forearm back), and back of the medial margin of the ulna, using stainless steel filiform needles which were disinfected (size: 1.5 inches, no. 30, Wujiang Jiachen Acupuncture Instrument Co., Ltd., China). The needle handle, holding by the thumb, index finger, and middle finger, with 15°–30° from the needle tip to the skin, was rapidly punctured into subcutaneous skin and then slowly moved into 1.4 inches along a straight line after narrowing the angle to the skin. The needle was kept for 30 minutes if the patient felt that there was no pain, limp and numb, and swelling. The treatment was performed once a day, with 10 times as a course. The interval is 3–5 days. Moxibustion (moxa cones, Chongqing Happyall Medical Equipment Co., Ltd., China) was applied to the acupoints, including the Jingbailao point, Dazhui point, Jianzhongshu point, and Zhongzhu point. Moxibustion was lasted for 30 minutes through rotating the moxa cones to prevent burn. The treatment was conducted once on alternate days and 5 days for a course. Hot compression with Chinese herbal medicine, which is good for improving blood circulation and dispersing stasis, was performed on the patients' neck. The Chinese herbal medicine used for hot compression was provided by the Institute of Traditional Chinese Medicine of our hospital, including monkshood, spatholobus stem, *Paeonia lactiflora*, and Caulis Sinomenii, with 30 g each, all of which were put together in a cloth bag after steaming to about 70°C in a steamer. The bag was quickly moved back and forth on the patient's neck and shoulder until bag's temperature dropped to about 50°C and then was applied to the neck and shoulder until heat disappeared. The treatment was maintained 15–30 minutes once, twice a day with 15 days as a cycle. During the treatment, the patients in the two groups got enough sleep and neck-shoulder-back muscle exercises. The efficacy was evaluated after 30 days.

### 2.3. Evaluation of Therapeutic Effectiveness

The criteria for efficacy evaluation are as follows: (a) full recovery is defined as disappearance of the lesion by imaging examination, complete regression of clinical symptoms such as pain and numbness, and normal cervical vertebra function; (b) remarkable response is considered as a significant improvement on the lesion by imaging examination, disappearance of clinical symptoms, and enhancement of cervical vertebra function (negative neck turning test); (c) good response is thought as an improvement on the lesion by imaging examination, remarkable alleviation of clinical symptoms, and slight improvement on cervical vertebra function (the patient can straighten and raise the neck by 10°); (d) nonresponse is defined as no significant change in the lesion by imaging examination and no remarkable improvement or even worse situation on clinical symptoms and cervical vertebra function.

### 2.4. Outcome Measures

Six aspects, including pain intensity, numbness intensity, concentration of plasma beta-endorphin (*β*-EP) and substance P (SP), cervical vertebra function, quality of life, and adverse reactions, are involved. Visual analogue scale (VAS) [12], Northwick Park Neck Pain Questionnaire (NPQ) [13], and McGill Pain Questionnaire (MPQ) [14] are used to assess pain intensity of the patients. The higher the score, the greater the pain intensity. Visual comparison method was applied to evaluate numbness intensity. In brief, a 10 cm straight line with a scale was used, and both ends represent nonnumbness and severe numbness, respectively. The score is converted into 0 to 10 points according to the scale marked by the patient. The higher the patient's score is, the more severity of numbness is. Before and after treatment, 4 ml fasting venous blood was extracted from each subject and placed into EDTA-contained tubes, and the plasma was centrifuged at 3000 r/min for 15 min. The concentrations of *β*-EP and SP were measured by the enzyme-linked immunosorbent assay (ELISA). The ET-1 and CGRP concentrations in the plasma were measured by the radioimmunoassay method using commercially available kits (Jiancheng Bioengineering Institute, Nanjing, China), following the manufacturer's instructions. Neck disability index (NDI) [15], consisting of 50 questions, with a total score of 0–50, is carried out to evaluate cervical vertebra function. The higher score patients get suggests the worse function. Short Form-36 (SF-36) health survey questionnaire [16] is used to reflect the quality of life, which involves 36 questions. The higher the score is, the less the impact on the ability of daily living is. The occurrence of adverse reactions during treatment in the two groups is recorded. A trained nurse conducted the survey and guided the patients to fill in the questionnaires with unified guidelines. After doing a good job of explanation, the patient shall fill in the form item by item, and the questionnaire shall be collected on-site.

### 2.5. Statistical Methods

SPSS 20.0 software is used to analyze the data. After normal distribution of data by Shapiro–Wilk test, the measurement data are expressed as mean ± standard deviation and analyzed by *t*-test. The counting data are examined by chi-square test. *P* < 0.05 indicates the difference which is statistically significant.

## 3. Results

### 3.1. Baseline Information of Study Subjects between the Two Groups

Included CR patients were assigned to the study group and control group randomly, with 41 cases in each group. There was no significant difference on gender, age, body mass index, and disease duration between the two groups (*P* > 0.05), which was comparable, as shown in Table 1.

### 3.2. Therapeutic Effectiveness between the Two Groups

The CR patients, who were supplemented with wrist-ankle acupuncture-moxibustion and hot compression with Chinese herbal medicine, recovered better than those who received standard treatment only. The total response rate in the study group and control group was 97.55% and 82.93%, respectively, indicating that the treatment of wrist-ankle acupuncture-moxibustion and hot compression of the traditional Chinese medicine bag is beneficial to recovery for the patients with CR (*P* < 0.05, Table 2).

### 3.3. Additional Wrist-Ankle Acupuncture-Moxibustion and Hot Compression with Chinese Herbal Medicine Alleviated Pain and Numbness for CR Patients

The pain intensity of all patients was assessed by the VAS, NPQ, and MPQ. Evaluation of numbness intensity was performed by the visual comparison method. According to the data, as listed in Table 3, we found that the patients in the study group obtained significant lower scores compared to the control group (*P* < 0.05), suggesting that the supplementation of wrist-ankle acupuncture-moxibustion and hot compression with Chinese herbal medicine contributes to alleviate pain and numbness of the neck.

### 3.4. Additional Wrist-Ankle Acupuncture-Moxibustion and Hot Compression with Chinese Herbal Medicine Reduced ET-1 and SP Concentrations and Increased CGRP and *β*-EP Concentrations

**Figure 1:**
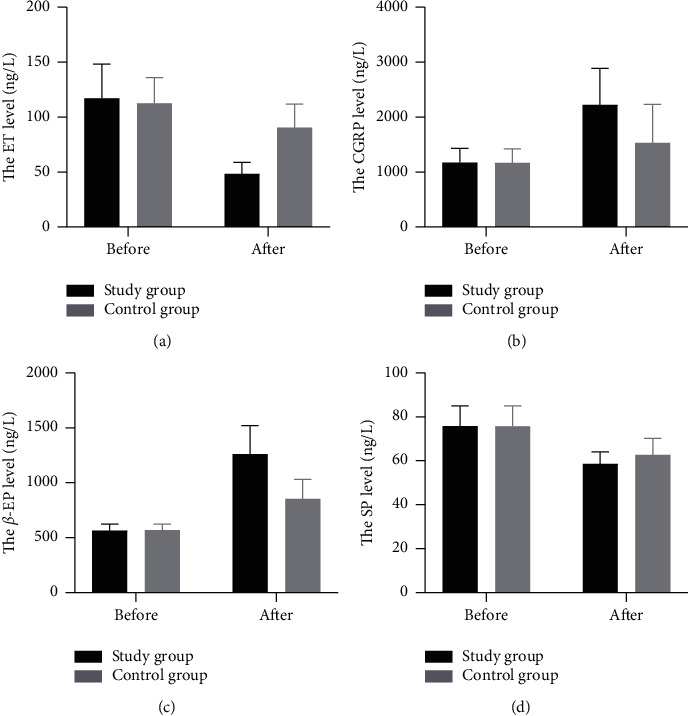
The plasma levels of ET-1, CGRP, *β*-EP, and SP in CR patients in the study and control groups after and before treatment.

ET-1 has been reported as a circulating factor that leads to vasoconstriction and is involved in the initiation of CR. Release of ET-1 inhibits CGRP production and following effects. As shown in Figure 1, the plasma level of ET-1 was reduced, but the plasma level of CGRP was elevated after treatment in both groups (*P* < 0.01). Notably, the CR patients in the study group showed a lower plasma level of ET-1 with a higher plasma level of CGRP than those in the control group (*P* < 0.01). ELISA was applied to detect the concentrations of plasma *β*-EP and SP before and after treatment. It was observed that the two groups showed a higher concentration of plasma *β*-EP but a lower concentration of SP. These data in the study group were superior than those in the control group (*P* < 0.05), which reveal that additional wrist-ankle acupuncture-moxibustion and hot compression with Chinese herbal medicine reduced ET-1 and SP concentrations and increased CGRP and *β*-EP concentrations, resulting in relief of pain.

### 3.5. Additional Wrist-Ankle Acupuncture-Moxibustion and Hot Compression with Chinese Herbal Medicine Improved Cervical Vertebra Function and Quality of Life

The cervical vertebra function was assessed by NDI. Quality of life was scored by SF-36. It was found that, after treatment, the NDI and SF-36 scores in the two groups manifested lower value and higher value, respectively. In addition, the patients in the study group were with a lower score of the NDI and higher score of SF-36 compared to the control group (*P* < 0.05, Table 4). These data indicated that cervical vertebra function was enhanced and quality of life was improved by the treatment of wrist-ankle acupuncture-moxibustion and hot compression with Chinese herbal medicine.

### 3.6. Occurrence of Adverse Reactions in the Two Groups

No obvious adverse reactions were found in the two groups during the treatment. After treatment, no apparent abnormalities such as routine blood examination, liver dysfunction, and kidney dysfunction occurred in the blood test.

## 4. Discussion

CR is caused by the compression or stimulation of nerve roots in the cervical spine and mainly characterized by arm pain and neck pain and sensory abnormalities in some cases leading to decreased muscle strength, sensory changes, and impaired deep tendon reflex, which is prevalent among people in their 50s. In recent years, research studies have shown that younger populations tend to suffer from CR due to lifestyle changes, and the prevalence rate is increasing year by year [17, 18]. Most patients with CR have been improved with nonsurgical intervention, involving physical therapy, traction, soft collar, massage, oral medications, and steroid injection [2]. However, little evidence from high-quality studies does not support any single treatment. Acupuncture, moxibustion, and hot compression with Chinese herbal medicine are essential parts of traditional Chinese medicine and have been widely applied to alleviate pain in clinical trials [19, 20]. However, the specific mechanism of the combination of acupuncture, moxibustion, and hot compression with Chinese herbal medicine in CR remains unclear. The study we presented aimed to analyze the efficacy of the combined treatment of wrist-ankle acupuncture, moxibustion, and hot compression with Chinese herbal medicine on the CR patients.

CR belongs to the category of “arthralgia disease” in traditional Chinese medicine. It is mainly caused by blood insufficiency and muscle strain, resulting in blocking main and collateral channels of the nape. Therefore, the treatment should pay attention to invigorating the circulation of blood, removing obstruction in the channels, and relaxing muscles. Wrist-ankle acupuncture, as a subcutaneous acupuncture technique, has manifested its alleviated function on chronic and acute pain without any needling sensation [21]. Moxibustion is a form of burning of herbal moxa cones, wormwood leaf, or mugwort over acupuncture points, which is associated with inflammatory pain relief [22], and it has also been turned out to be an effective intervention in menstrual pain relief [23]. The hot compression with Chinese herbal medicine has improved cervical range of motion as well [19]. These findings were similar to ours. In terms of total response rate, the study group revealed significantly higher rate than the control group, indicating that wrist-ankle acupuncture combined with moxibustion and hot compression with Chinese herbal medicine is effective in the treatment of CR, which might be due to its function of pain relief, vasospasm relief, and blood circulation improvement.

In order to assess the pain intensity and numbness intensity in CR patients, various tests were performed including VAS, NPQ, MPQ, and visual comparison method. It was observed that the patients, who received wrist-ankle acupuncture combined with moxibustion and hot compression with Chinese herbal medicine, in the study group obtained significant lower scores compared to the control group. Likert-type scale is one of the most commonly used scoring summation scales, which measures characteristics of participants. Typically, it has more than two options to choose from. The participants choose the one that best reflects their status and characteristics [24]. VAS has been a common and effective tool for measuring pain, quality of life, and anxiety since the 1920s [25]. NPQ is one of the most frequently used indicators to evaluate patients' neck dysfunction [26]. MPQ and short-form MPQ have been extensively applied to the clinical pain studies, which are of great significance in the study of stimulating pain perception [27]. In addition, this study evaluated cervical vertebra function through NDI and measured quality of life by SF-36. NDI is the first tool designed to assess patients' neck dysfunction [28]. SF-36 is an instrument for assessment in health status. It has been confirmed that the diabetic patients were with a lower score of SF-36 [29]. We found that the patients in the study group were with a lower score of NDI and higher score of SF-36 compared to the control group, manifesting neck dysfunction, and quality of life was improved after treatment of wrist-ankle acupuncture-moxibustion and hot compression with Chinese herbal medicine. In another CR study, Ding et al. pointed out that traditional Chinese medicine hot compression combined with traction was related to pain relief reflecting from the VAS and NDI [30].

To confirm the effectiveness of wrist-ankle acupuncture-moxibustion and hot compression with Chinese herbal medicine in treating CR, the plasma concentrations of ET-1 and CGRP in patients with CR were examined. Our study demonstrated that the CR patients in the study group showed a lower plasma level of ET-1 with a higher plasma level of CGRP than those in the control group. ET-1 is a family of peptides that are produced primarily by vascular endothelial cells and was discovered in 1980. It was regarded as a vasoconstrictor and mitogen for smooth muscles (PMID: 33369150). ET-1 binds to two types of receptors, ETA and ETB. The receptor ETA is localized on smooth muscle cells, while the receptor ETB is found in both endothelial cells and smooth muscle cells. Activation of ETA and ETB on smooth muscle cells affects the vasoconstrictive and mitogenic effects of ET-1. ETB stimulation in the endothelial cells increases the clearance of ET-1 and activation of NO and prostacyclin release. Endothelial cells release a large amount of ET-1 into blood, acting on the cerebellum and brainstem, leading to more severe ischemia and hypoxia in the vertebral artery, suggesting that ET-1 might be involved in the progression of CR. CGRP has roles in regulating the function of components of the immune system including dendritic cells, endothelial cells, T cells, B cells, and mast cells [31]. Cervical instability leading to the stimulation of the sympathetic fibers promotes the release of neuropeptide Y in the plasma and reduces the synthesis and release of CGRP by presynaptic regulation of the cardiac sensory nerves. In addition, ET production after sympathetic nerve excitation also inhibited the release and biological effects of CGRP [32]. Furthermore, we conducted ELISA to detect the concentration of plasma *β*-EP and SP before and after treatment. *β*-EP is a peptide that plays a variety of roles in the whole body. It has been found to have strong analgesic activities [33]. SP is a peptide mainly generated by neurons, which is involved in many biological processes, such as injury and inflammation [34]. Eyigör et al. revealed that the patients with mucosal contact headache had a higher concentration of SP than healthy individuals [35]. The ELISA tests indicated that compared to the control group, the patients in the study group had a higher concentration of *β*-EP and lower concentration of SP. As for the adverse reactions in the two groups, there were no obvious adverse reactions during the treatment and after treatment.

In summary, the regular anti-inflammatory and analgesic drugs could alleviate pain and numbness in CR patients. However, the supplementation of wrist-ankle acupuncture-moxibustion and hot compression with Chinese herbal medicine achieves better efficacy through promoting *β*-EP release and inhibiting SP production. It has an excellent response in relieving pain, restoring cervical vertebra function, and improving the quality of life. The deficiency of this study is that the included sample size is small, which may cause some statistical errors to the results. In addition, as to wrist-ankle acupuncture treatment, many factors may contribute to variability in efficacy, such as acupuncture technique, number of needles used, duration of needle retention, and psychological factors.

## Figures and Tables

**Table 1:** Basic information of the patients in the two groups.

Group	*n*	Gender		Age (year)	Body mass index (kg/m^2^)	Course of the disease (years)
		Male (*n*, %)	Female (*n*, %)			
Study group	41	22 (53.66)	19 (46.34)	53.26 ± 5.58	21.52 ± 1.42	5.38 ± 1.45
Control group	41	24 (58.54)	17 (41.46)	52.93 ± 5.72	21.63 ± 1.58	5.63 ± 1.12
*χ* ^*2*^ */t*		0.198		0.264	0.332	0.874
*P*		0.656		0.792	0.741	0.385

**Table 2:** Primary outcomes of patients after treatment between the two groups.

Group	*n*	Full recovery (*n*, %)	Remarkable response (*n*, %)	Good response (*n*, %)	Nonresponse (*n*, %)	Total response rate (*n*, %)
Study group	41	23 (56.10)	9 (21.95)	8 (19.51)	1 (2.44)	40 (97.55)
Control group	41	12 (29.27)	13 (31.70)	9 (21.95)	7 (17.07)	33 (82.93)
*χ* ^*2*^						4.986
*P*						0.026

**Table 3:** Scores of pain and numbness intensity between the two groups.

Group	*n*	Time	VAS	NPQ	MPQ	Numbness intensity
Study group	41	Before treatment	6.64 ± 1.18	39.41 ± 5.56	16.31 ± 2.24	5.79 ± 1.26
		After treatment	1.78 ± 0.49	14.09 ± 2.01	3.69 ± 0.75^ab^	1.84 ± 0.33^ab^

Control group	41	Before treatment	6.57 ± 1.51	39.57 ± 5.18	16.28 ± 2.37	5.83 ± 1.42
		After treatment	2.45 ± 0.62	19.31 ± 2.39	5.58 ± 1.16^a^	3.65 ± 0.68^a^

VAS: visual analogue scale; NPQ: Northwick Park Neck Pain Questionnaire; MPQ: McGill Pain Questionnaire. ^a^indicated the data were statistically significant compared with that before treatment. ^b^demonstrated the data were statistically significant compared with that of control group.

**Table 4:** NDI and SF-36 scores between the two groups.

Group	*n*	Time	NDI	SF-36
Study group	41	Before treatment	15.79 ± 2.52	100.76 ± 8.28
		After treatment	6.68 ± 1.31^ab^	116.77 ± 11.65^ab^

Control group	41	Before treatment	16.15 ± 2.47	101.58 ± 8.56
		After treatment	9.16 ± 1.45^a^	110.36 ± 10.35^a^

NDI: neck disability index; SF-36: Short Form-36 health survey questionnaire. ^a^indicated the data were statistically significant compared with that before treatment. ^b^demonstrated the data were statistically significant compared with that of control group.

## Data Availability

The data used to support the findings of this study are included within the article.
